# A 90‐channel triaxial magnetoencephalography system using optically pumped magnetometers

**DOI:** 10.1111/nyas.14890

**Published:** 2022-09-05

**Authors:** Molly Rea, Elena Boto, Niall Holmes, Ryan Hill, James Osborne, Natalie Rhodes, James Leggett, Lukas Rier, Richard Bowtell, Vishal Shah, Matthew J. Brookes

**Affiliations:** ^1^ Sir Peter Mansfield Imaging Centre, School of Physics and Astronomy University of Nottingham Nottingham UK; ^2^ QuSpin Inc. Louisville Colorado USA

**Keywords:** electrophysiology, human brain imaging, magnetoencephalography, optically pumped magnetometers

## Abstract

Magnetoencephalography (MEG) measures the small magnetic fields generated by current flow in neural networks, providing a noninvasive metric of brain function. MEG is well established as a powerful neuroscientific and clinical tool. However, current instrumentation is hampered by cumbersome cryogenic field‐sensing technologies. In contrast, MEG using optically pumped magnetometers (OPM‐MEG) employs small, lightweight, noncryogenic sensors that provide data with higher sensitivity and spatial resolution, a natural scanning environment (including participant movement), and adaptability to any age. However, OPM‐MEG is new and the optimum way to design a system is unknown. Here, we construct a novel, 90‐channel triaxial OPM‐MEG system and use it to map motor function during a naturalistic handwriting task. Results show that high‐precision magnetic field control reduced background fields to ∼200 pT, enabling free participant movement. Our triaxial array offered twice the total measured signal and better interference rejection compared to a conventional (single‐axis) design. We mapped neural oscillatory activity to the sensorimotor network, demonstrating significant differences in motor network activity and connectivity for left‐handed versus right‐handed handwriting. Repeatability across scans showed that we can map electrophysiological activity with an accuracy ∼4 mm. Overall, our study introduces a novel triaxial OPM‐MEG design and confirms its potential for high‐performance functional neuroimaging.

## Graphical abstract

Magnetoencephalography (MEG) measures the tiny magnetic fields generated by current flow in the brain, but conventional MEG is hampered by cumbersome cryogenic field‐sensing technologies. Our wearable, 90‐channel triaxial optically‐pumped magnetometer (OPM) MEG system—in conjunction with precision magnetic field control that reduced background fields to ∼200 pT—is used here to map electrophysiological activity during handwriting to the motor cortex, with accuracy ∼4 mm.

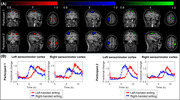

## INTRODUCTION

Magnetoencephalography using optically pumped magnetometers (OPM‐MEG) is a new way to noninvasively assess human brain activity. Like conventional MEG, the technique measures magnetic fields generated above the scalp by current flow in assemblies of neurons.^1^, ^2^, ^3^ These fields, with appropriate mathematical analysis, allow us to infer moment‐to‐moment changes in electrical brain activity as a participant carries out a task.^4^ However, unlike conventional MEG, which employs a fixed array of cryogenically cooled magnetic field sensors (i.e., superconducting quantum interference devices—SQUIDs), OPM‐MEG uses a new generation of sensors (OPMs) that are small and lightweight, do not require cryogenic cooling, and can be mounted flexibly on or near the scalp.^5^ The result is a more practical scanner that is free from cryogenics and can, in principle, adapt to different head sizes and shapes, enabling free movement during scanning.^5^, ^6^, ^7^, ^8^, ^9^ Since less thermal insulation is required between the sensor and the head, the OPMs are positioned closer to the brain (compared to conventional SQUID‐based systems), thereby improving sensitivity and spatial precision.^10^, ^11^, ^12^, ^13^ The potential of OPM‐MEG as a high‐performance functional imaging modality is significant, and growing enthusiasm within the neuroscientific and clinical research communities has been seen in recent years. However, OPM‐MEG is a nascent technology, and there are significant challenges to the successful implementation of an optimized system, including control of background magnetic fields, the design of OPMs, and implementation of sensor arrays. Here, we bring together new developments in each of these key areas to demonstrate how a novel, 90‐channel OPM‐MEG instrument can accurately characterize brain function during a naturalistic task.

OPMs rely on the quantum properties of alkali atoms (e.g., ^87^Rb) to detect magnetic fields.^5^ Briefly, an atomic vapor housed in a glass cell is illuminated with a laser. If the wavelength of the laser is resonant with the D1 transition of the atomic species, the atoms absorb photons from the laser and, in an absolute zero magnetic field, are optically pumped into a "dark state". Atoms in a dark state cannot absorb more photons making the vapor transparent to incident light.^14^ Alkali atoms possess a magnetic moment and, in the absence of a magnetic field, the effect of pumping is to align those magnetic moments along the direction of the laser. Consequently, the vapor gains a *bulk* magnetic moment (i.e., the sum of all individual magnetic moments pointing in the same direction). Once aligned and because the vapor is transparent, the intensity of laser light passing through the cell is maximized (known as a zero‐field resonance). However, in the presence of an external magnetic field, the bulk magnetization is deflected away from alignment. The atoms can once again absorb photons, and the light passing through the cell is reduced. The intensity of light passing through the vapor thus becomes a function of the magnetic field, and the system behaves as a magnetometer.^15^ In practice, this mode of operation is complicated by atomic collisions (which reduce bulk magnetization) and by first‐order insensitivity of the transparency to field orientation. However, operation in the spin‐exchange relaxation‐free (SERF) regime^16^ and application of a known oscillating field across the cell^17^ counteracts these effects—enabling magnetic fields to be sampled accurately with directional sensitivity in two orthogonal orientations, perpendicular to the laser.

**FIGURE 1:**
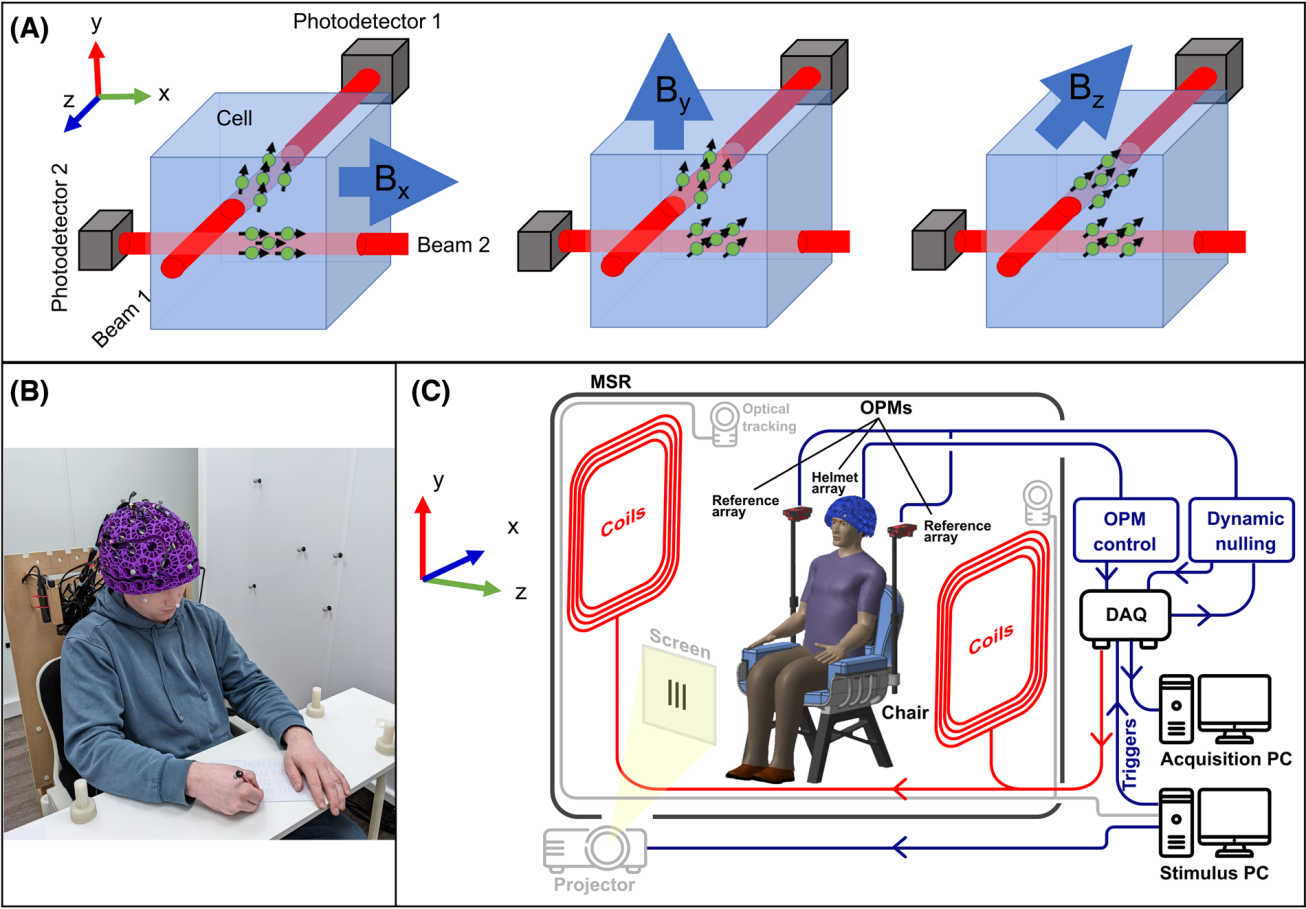
System overview. (A) Schematic diagrams of a triaxial OPM. Two independent laser beams are projected through the vapor cell. The beam oriented in *z* allows measurements of the field components B_x_ and B_y_. The beam oriented in *x* allows measurements of the field components B_z_ and B_y_. These four measurements can be combined to determine the full vector magnetic field. Note, however, that B_y_ is measured twice and thus has marginally lower noise. (B) Photograph of a participant wearing the OPM‐MEG helmet, taking part in the handwriting paradigm. (C) A schematic diagram of the OPM‐MEG system. The participant is placed in a magnetically shielded enclosure (in this case, a room of internal dimension 3 × 2.4 × 3 m^3^, whose walls comprise four layers of mu‐metal and a single layer of copper). Biplanar coils are placed on either side of the participant for background field control. Data acquisition and storage, as well as coil control, are implemented via digital acquisition systems coupled to a PC. A second PC controls the experimental paradigm and motion tracking.

OPMs are well established as a successful means to measure the MEG signal; however, this mode of operation offers significant flexibility for OPM design, and the optimal sensor configuration is not yet set. One recent development is the triaxial OPM, which can simultaneously measure magnetic field components along three orthogonal orientations.^30^ This is made possible via the use of two laser beams. Specifically (assuming Cartesian coordinates), a beam oriented in *x* enables field measurement in *y* and *z*; a second beam, oriented in *z*, enables field measurement in *x* and *y*. Combining all four measurements allows us to determine the full vector field (Figure 1A). Triaxial measurement of the magnetic field is promising for a number of reasons. First, almost all conventional MEG systems measure a single component of the field vector (radial to the head surface). Triaxial OPMs provide two additional (tangential) metrics and thus three times more measurements. The tangential field components generated by neural sources are smaller in magnitude than the radial components^12^, ^31^, ^32^ so they do not equate to three times more signal. Nevertheless, an array containing 50 triaxial sensors is approximately equivalent to an array of 80 conventional (radial) sensors in terms of total signal acquired.^32^ Second, theory shows that triaxial measurement is better than radial‐only measurements for differentiating fields originating inside the head (i.e., the MEG signal) from fields originating in the environment (i.e., interference). This provides a marked advantage in terms of signal quality since artifacts can be identified and rejected based on the spatial signature of their magnetic field.^32^ Third, when the number of sensors is limited (which is usually the case), triaxial measurement offers advantages in terms of the uniformity of coverage—particularly in the case of pediatric measurements.^31^, ^33^ Finally, the ability to characterize the complete field vector offers significant advantages for sensor calibration, elimination of crosstalk (both between axes in a single sensor and between adjacent sensors), and the prospect of robust “closed‐loop” operation, whereby sensors are operated in a continuous feedback loop to improve dynamic range in all three directions. Evaluation of a small number of triaxial OPMs for MEG has been undertaken,^33^ and the sensitivity of these prototypes was similar to the more conventional single‐ and dual‐axis OPMs (triaxial sensitivities of ∼9–14 fT/sqrt (Hz) compared to ∼7–10 fT/sqrt (Hz)), potentially making triaxial sensors well suited for MEG. However, at the time of writing, to our knowledge, no one has built a large‐scale triaxial array.

Aside from OPM and array design, the biggest challenge in OPM‐MEG—particularly if free participant movement is to be enabled—is control of the background magnetic field. Magnetic fields from the brain are ∼100–1000 fT in amplitude; several orders of magnitude smaller than naturally occurring environmental fields. For this reason, all systems (including cryogenic MEG) are housed within a magnetically shielded environment (MSE) constructed using multiple layers of high magnetic permeability and high electrical conductivity metals. These layers reduce temporally fluctuating environmental fields at low and high frequencies, respectively, to a level where brain activity can be delineated from background fluctuations. However, in such environments, the presence of metal in the walls leaves a remnant static (i.e., constant over time) magnetic field of 20‒70 nT. This field does not affect cryogenic sensors; however, it is too large for an OPM to achieve its zero‐field resonance and OPMs will not function. Once in operation, the dynamic range of an OPM (i.e., the range of magnetic field it can accurately measure) is small (∼±3 nT for gain errors less than 5%), meaning that movement of the sensor with respect to the static field causes a measurable change in the field that may exceed its dynamic range. This is a key consideration given that one of the major advantages of OPM‐MEG is free participant movement. For these reasons, both the static magnetic field and temporally fluctuating fields must be controlled in OPM‐MEG.

In practice, electromagnetic coils can be placed inside each OPM to cancel the static magnetic field at each sensor. Energizing these *on‐sensor* coils at the start of a MEG experiment will enable operation if the OPM remains stationary.^10^ However, if the participant moves, the OPM measures a change in the magnetic field that may exceed its dynamic range (e.g., a 4° rotation in a 30 nT field would be sufficient to prevent the OPM working). There are two solutions to this. The sensor can be operated in a *closed‐loop* configuration, whereby the on‐sensor coils are continually updated based on the sensor read‐out in order to keep the field within the OPM close to zero.^34^ In principle, this would allow the OPM to operate even if it were moving through a large magnetic field. However, closed‐loop sensing preserves artifacts generated by movement, causes sensors in close proximity to interfere with one another,^34^ and introduces implementation challenges because the shape of the zero‐field resonance is strongly affected by fields in all three orientations. An alternative solution is to remove the static field entirely by using reference sensors inside the MSE in conjunction with large electromagnetic coils situated around the participant. A feedback loop, alongside judicious coil design, enables both spatially uniform magnetic fields and linear field gradients to be compensated across the entire head.^35^, ^36^, ^37^ Removal of the field in this way not only ensures OPMs remain operational when moving but also reduces movement artifacts. Such systems have proved effective—enabling the construction of a wearable system where participants have been able to move during a scan.^6^, ^9^, ^35^, ^38^ However, some degree of remnant magnetic field remains, and it is not yet known whether a genuine zero‐field could be achieved with such a system.

In this paper, we bring together novel triaxial OPMs with newly developed magnetic shielding techniques to demonstrate a 90‐channel OPM‐MEG system operating in a close‐to‐zero‐field environment. To demonstrate effectiveness, we employ an experimental paradigm in which participants write down a word shown on a screen with either their left or right hand. Using this task, we elicit known effects associated with movement (specifically the event‐related beta decrease, the postmovement beta rebound,^39^, ^40^ and their modulation with left‐ and right‐handed movements). We hypothesized that these well‐characterized effects could be measured with high fidelity using our system, despite the natural, large‐scale head movements required to perform the task.

To assess repeatability between scans, we measure both neural oscillatory modulation and electrophysiological functional connectivity in the same participants multiple times. We localize the maximum amplitude modulation in the beta‐band for each task condition and quantitatively assess repeatability by comparing this localization across repeat experiments. To further assess system robustness, we exploit a phenomenon known as *neural fingerprinting*, where it has been shown^41^, ^42^, ^43^ that brain activity is unique to an individual (e.g., a fingerprint). We measure the correlation between repeat experiments for functional images and connectome graphs, and we hypothesize that assuming our system provides high‐fidelity data in moving participants, within‐participant correlations should be higher than between‐participant correlations. Finally, we test the extent to which background magnetic fields can be suppressed using a field nulling system and analyze the advantages of MEG reconstruction using a triaxial array.

## METHODS

### System overview

Thirty OPMs with triaxial sensitivity^30^, ^33^ (QuSpin Inc., Louisville, CO, USA) were operated in a single array. The sensors were housed in a 3D‐printed helmet (Cerca Magnetics Ltd., Nottingham, UK) worn by the participant (Figure 1B). This helmet can house up to 64 OPMs; the 30 sensors available were arranged to provide coverage of the left and right sensorimotor cortices. The participant was seated at the center of a 3 × 2.4 × 3 m^3^ (internal dimensions) magnetically shielded room (MuRoom, Magnetic Shields Ltd., Kent, UK), and between a set of biplanar electromagnetic coils (Cerca Magnetics Ltd.) that were used for background magnetic field control. The participant's head is positioned in the central volume between the coil planes. The coils themselves are 1.6 × 1.6 m^2^ in area, with a separation of 1.5 m. Four additional OPMs (first generation, dual‐axis, QuSpin Inc.) were placed behind the participant to measure field fluctuation inside the MSE. A set of six infra‐red (IR) cameras (OptiTrack Flex 13, NaturalPoint Inc., Corvallis, OR, USA) were placed around the participant and used to optically track the position of IR‐retroreflective markers, attached to the OPM‐MEG helmet and biplanar coils. A schematic diagram of the system is shown in Figure 1C. The outputs of OPMs in both the helmet and reference arrays are fed into a digital acquisition (DAQ) system (National Instruments, Austin, TX, USA) and recorded by a computer. An additional DAQ is used to control the currents through each of the electromagnetic coils via a set of low‐noise current drivers (QuSpin Inc.). A single PC controlled the OPMs, coils, data acquisition, and storage, while a separate PC controlled the experimental paradigm (i.e., what the participant sees throughout the experiment) and the motion tracking cameras.

### Paradigm

Two participants took part in the study. Both gave written, informed consent, and the study was approved by the University of Nottingham Medical School Research Ethics Committee. The participants (henceforth termed 1 and 2) were 42 and 27 years old; both were male and right‐handed (both scored 100 on the Edinburgh handedness test).

The paradigm was implemented using custom code written in Presentation (Neurobehavioral Systems Inc., Berkeley, CA, USA). A single experiment comprised two blocks of 20 trials. In a single trial, a randomly selected five‐letter word was presented on a screen for 5 s; this was followed by a 7 s rest period (indicated by a blank screen). Upon stimulus presentation, the participant wrote down the word shown using a pencil and paper. In one block of trials, the participant used their left hand to complete the task, and in the other block, they used their right hand. An instruction telling the participant which hand to use was shown on the screen at the start of each block. The words were selected at random from a database of 200. Within a single experiment, the words, and the order in which they were presented, were the same for both blocks (i.e., participants wrote the same words, in the same order, with their left and right hand). However, the words differed between experiments. Both participants undertook this experiment eight times, and the order in which the left‐ and right‐handed blocks were presented was alternated between runs.

### Background magnetic field compensation

For each of the 16 experiments, compensation of both static and time‐varying magnetic fields in the region containing the OPM‐MEG helmet was undertaken based on a technique originally described by Rea et al.^37^ Compensation fields were generated using the biplanar coils shown in Figure 1C. This coil system is designed to output the three homogeneous magnetic field components and all five independent (linear in space) field gradient components. All coils had previously been calibrated to determine the magnetic field (or field gradient) per unit current generated by each coil. The procedure of magnetic field compensation was as follows.

Immediately following the positioning of the participant in the center of the MSE and coils, the MSE door was closed and the inner mu‐metal panels in the walls demagnetized.^44^ This optimizes the performance of passive shielding, leaving (on average) around a 3‒5 nT spatially homogeneous field in the center of the room with field gradients of order 2‒4 nTm^−1^. This procedure takes approximately a minute to complete.

Following demagnetization, we aimed to compensate for any (slow) drift in the magnetic field over time using dynamic stabilization.^36^ Such drift can result from, for example, temperature variations causing small changes in the spatial dimensions of the MSE. The OPM reference array was used to sample changes in the magnetic field over time. The outputs of these sensors were low‐pass filtered at 3 Hz and input to a high‐speed (60 Hz) proportional integral controller, implemented in LabVIEW (National Instruments), and used to determine compensation currents, which were applied dynamically to the biplanar coils. This approach stabilizes <3 Hz changes in the three uniform components of the magnetic field, and the three gradients that vary in *z*. (Note: the reference sensors were separated by ∼40 cm in *z*, but had similar *x*‐ and *y*‐coordinates so dynamic stabilization of gradients varying in the *x*‐ and *y*‐directions was not possible using this arrangement.)

Having applied dynamic stabilization, the background magnetic field was held constant over time; however, the temporally static offset field (i.e., the spatially homogenous field and associated gradients that exist following demagnetization) remained. This was sampled by moving the helmet through the background field and simultaneously recording the resulting change in magnetic field measured at the OPMs along with the helmet location. Specifically, the participant was asked to complete a series of head movements for 60 s—rotating and translating their head about each of the three Cartesian axes to sample the magnetic field and its spatial variation, comprehensively. Throughout this process, the location and orientation of the rigid helmet with respect to the coils were tracked using the six optical tracking cameras. This was achieved by attaching five markers to known positions on the helmet and five markers to one coil plane as a stationary reference. The locations and orientations of the sensors, relative to the helmet, were known as a result of the 3D printing process. The magnetometer and corresponding motion data derived from this procedure were then fit to a spherical harmonic model comprising the eight components of the magnetic field (i.e., three spatially uniform magnetic field components and five field gradient components) that can be generated by the coils. This modeling, combined with the coil calibration data, enabled us to apply DC currents to the coils such that an equal and opposing magnetic field was generated to cancel the remnant field. Having applied the currents, the final magnetic field should be close to zero across the volume occupied by the OPM helmet. Dynamic stabilization remains active through data acquisition to compensate for changes in the background field over time.

**FIGURE 2:**
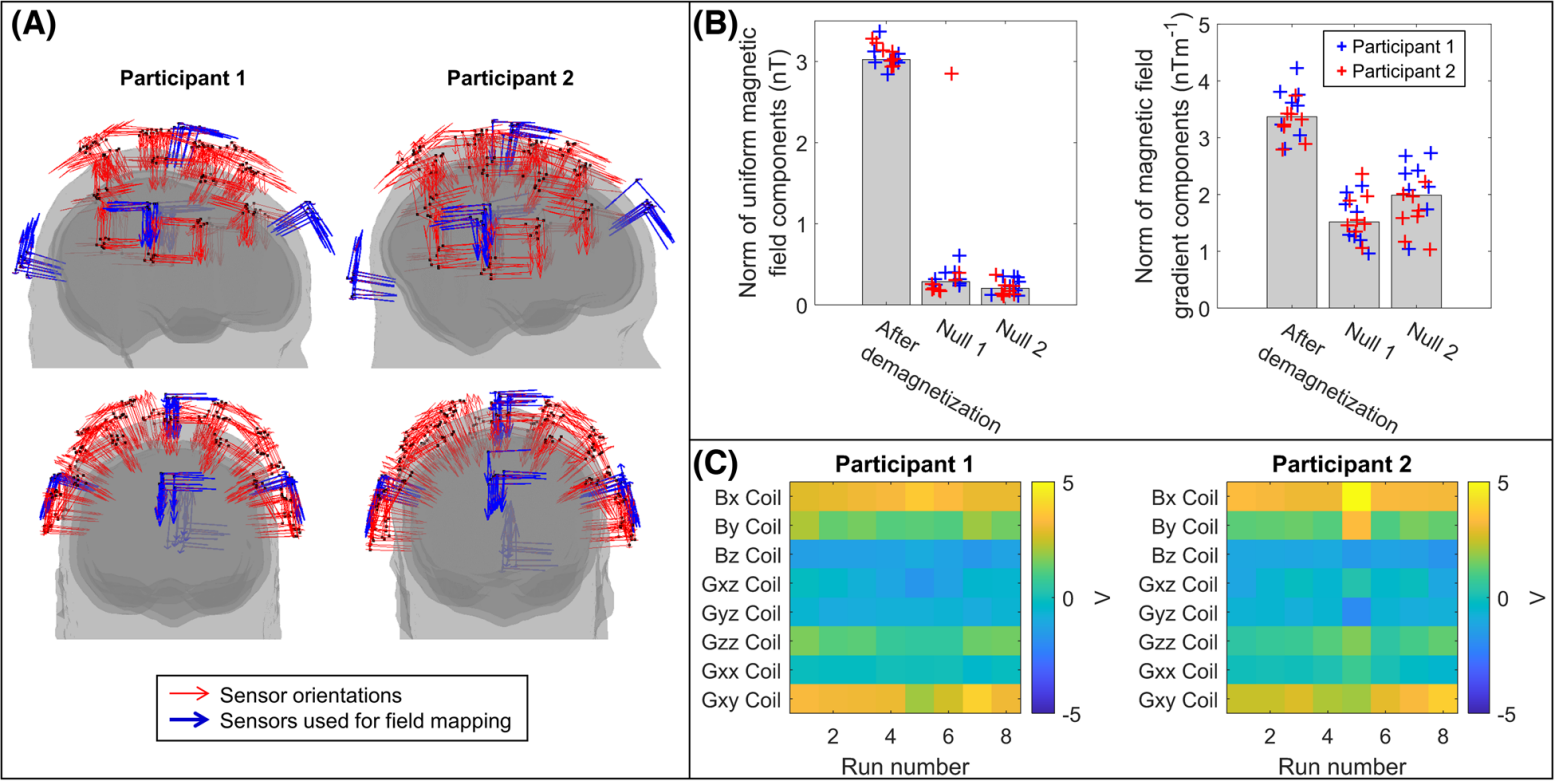
Background magnetic field control. (A) Sensor locations, registered to brain anatomy, for each run. Note: Although the helmet was in a slightly different position on the head in each experiment, the sensor positions and orientations are reasonably consistent. The five sensors used in the nulling procedure are shown in blue. (B) The norm of the three uniform magnetic field components, as determined by the model, is shown on the left. Values immediately following demagnetization and after two iterations of nulling are shown. Bars represent the median value across all 16 scans, while the individual data points are shown as blue crosses for participant 1 and red for participant 2. The norm of the five linear magnetic field gradient components is shown on the right. (C) Matrices showing the consistency of the final voltages applied to each of the coils across the eight scans for each participant.

The process of field mapping, which included calculation and application of nulling currents, was performed twice to optimize the static magnetic field compensation for each experiment. The field mapping procedure was then undertaken a third time to capture the final field in which the helmet was situated. This procedure takes approximately 10 min. To simplify the fitting procedure and minimize scan time, triaxial measurements from only five of the 30 OPMs were used to fit the background magnetic field. These OPMs were positioned at the front, back, top, left, and right of the helmet (Figure 2A).

### Data acquisition

Ninety channels of OPM data were recorded throughout the handwriting paradigm, at 16‐bit resolution and a sampling rate of 1200 Hz.

The optical tracking system was used to measure both participant head motion and the location of the pencil throughout all experiments with six degrees of freedom. These data were recorded using the NaturalPoint Motive software platform at a sampling rate of 120 Hz. Control of Motive from MATLAB (MathWorks Inc., Natick, MA, USA) was facilitated by the NaturalPoint NatNet SDK software.

Synchronization of the magnetometer and motion data was achieved using a trigger channel controlled via MATLAB and recorded using the DAQ to indicate when the motion recording began. Additional triggers were recorded upon stimulus onset and presentation of the left‐ and right‐hand instructions in order to enable data segmentation.

Upon completion of the paradigm, the position of each OPM and the orientations of its sensitive axes were determined relative to the participant's brain anatomy by a coregistration procedure.^45^, ^46^ Three‐dimensional (3D) structured light scans (Structure Core, Occipital Inc., Boulder, CO, USA) were acquired, showing the location of the helmet relative to the participant's facial features. These data were aligned with the participant's anatomical MRI (acquired using a Phillips 3T Ingenia MRI scanner, at 1 mm isotropic resolution) and the known structure of the OPM‐MEG helmet (from 3D printing) in order to generate a complete, 3D registration of the sensor locations and orientations relative to the brain anatomy (Figure 2A).

The eight repeat experiments for each participant were conducted across 3 days and each run was distinct. Runs consisted of not only separate data acquisitions, but the participant also removed the helmet and left the room. Furthermore, the MSE walls were demagnetized, and separate coregistration procedures were undertaken.

### Data preprocessing

For each experiment, every channel of OPM data was mean corrected and segmented into left‐ and right‐handed blocks. All data were inspected visually, and no trials were rejected. Across the 16 experiments, one axis of one sensor failed in all recordings. During one recording in participant 2, all three axes of that same OPM failed. This sensor was, therefore, excluded from all analyses. This meant that 87 of the 90 channels collected were available for analysis. All three axes of measurement for each OPM were used in the analyses described below unless otherwise stated.

### Source localization

A beamformer was applied to left‐ and right‐handed trials separately using custom code written in MATLAB. In each case, data from all 87 OPM channels were filtered to the beta‐band (13–30 Hz) using a fourth‐order Butterworth filter. Data covariance matrices were generated (separately for each task condition) and regularized using the Tikhonov method with the regularization parameter set at 5% of the maximum eigenvalue of the unregularized matrix. Current sources in the polar and azimuthal orientations were reconstructed. The radial direction was ignored since MEG is relatively insensitive to radially oriented dipoles. The radial and tangential orientations were defined relative to a sphere that best fit the participants’ head shape. The beamformer weights were computed using a forward model that characterizes a current dipole in a single‐shell conductor^47^ implemented in FieldTrip.^48^ Based on the two reconstructed, tangential projections of current, the single orientation of maximum signal amplitude was computed using the method described by Sekihara et al.^49^

A functional image was constructed using a pseudo‐T‐statistical approach^50^ to show the location of current sources exhibiting the maximum beta amplitude modulation between active (1.5 s < *t* < 3.5 s relative to stimulus onset) and control time windows. The control time window was chosen to coincide with the postmovement beta rebound, whose timing varied between left‐ and right‐handed trials (9 s < *t* < 11 s and 5.5 s < *t* < 7.5 s, respectively), since, on average, it took both participants longer to write with their nondominant (left) hand. The brain anatomy was divided into regular 4 mm voxels for computation of the pseudo‐T‐statistic. For each of the 16 experiments, we calculated two pseudo‐T‐statistical images showing modulation in beta activity for left‐ and right‐handed writing, respectively.

To assess localization accuracy across runs in detail, these pseudo‐T‐statistical images were recomputed using 1 mm voxels, and the location (in mm) of the voxel with maximum beta amplitude modulation in the right and left hemispheres for left‐ and right‐handed writing, respectively, was determined for each run. The location of peak activation for each condition was averaged across runs for each participant, and the mean Euclidean distance between this average location and each of the eight individual peak locations for each run was determined.

### Visualization of neural activity

To visualize brain activity, we used a “virtual electrode” analysis to assess beta envelope modulation. Beamformer weights for a location of interest were calculated (as used above) and applied to the beta‐band filtered OPM data. The Hilbert envelope of the resulting signal was calculated and averaged across trials with the left‐ and right‐handed task conditions analyzed separately. This resulted in a time course of beta modulation throughout the trial.

In addition to the beta envelope, a trial‐averaged time‐frequency spectrum (TFS) was computed to show the evolution of oscillatory activity during the trial. To do this, reconstructed time courses (using beamformer weights calculated in the broad band) were filtered in a series of overlapping frequency bands, and the Hilbert envelope was computed for each band. Envelopes were then averaged over trials and concatenated in frequency to generate the TFS. TFS data were computed independently for both task conditions (i.e., left‐ and right‐handed writing) in each of the 16 experiments.

### Connectivity analysis

In addition to activity, we aimed to investigate functional connectivity specifically by examining: networks of brain regions that work in concert during the handwriting task, how those networks differ between left‐ and right‐handed writing, and whether these networks differ between individuals.

To compute a whole‐brain connectome, a brain parcellation was performed. For each participant, the brain anatomy was segmented into 78 cortical regions according to the automated anatomical labeling (AAL) atlas.^51^ Connectivity was estimated using amplitude envelope correlation, applied within the brain parcellation. A time course of beta‐band activity at the center of mass of each AAL region was extracted using the beamformer approach described above. This resulted in 78 regional time courses. Connectivity was assessed between every possible pair of regions resulting in 3003 independent functional connectivity measurements. One confound in MEG connectivity estimation is that due to the ill‐posed inverse problem, estimates of activity from spatially separate locations are not necessarily independent; rather, the signal from one region can leak into a neighboring region. Thus, for each region pair, we applied a pairwise orthogonalization whereby the beta‐band time course from region A was regressed from region B. This technique has been shown to effectively mitigate signal leakage.^52^, ^53^ Following this, the envelope of beta oscillations was computed using a Hilbert transform and downsampled to 10 Hz. The Pearson correlation between envelope time courses was used as a metric of functional connectivity. Sequential application of this procedure for all possible region pairs resulted in a 78 × 78 connectome matrix with diagonal symmetry. These matrices were derived independently for left‐ and right‐handed writing—resulting in 32 independently computed connectomes for each run in each participant.

### Differences between left‐ and right‐handed writing

As an initial test of the system, we aimed to show a difference between left‐ and right‐handed writing. To this end, we undertook two separate statistical tests. First, we tested the hypothesis that beta modulation measured in the left and right sensory cortices would differ between task conditions. We reconstructed beta envelopes (as performed above) in both left and right sensory cortices (defined according to the AAL atlas) for all eight experiments in both participants. The magnitude of beta modulation was quantified as the difference in amplitude between an active and control window. Relative to stimulus onset for right‐handed writing, the active window was defined as 2 s < *t* < 4 s, and the control window was defined as 5 s < *t* < 7 s. These values were changed to 4 s < *t* < 6 s for the active window and 8 s < *t* < 10 s for the control window in left‐handed trials to account for slower writing. This process resulted in 32 measurements per participant (i.e., eight runs with estimates in two locations for the two conditions). We carried out independent Wilcoxon rank sum tests contrasting beta modulation between conditions for each location. We controlled for multiple comparisons using Bonferroni correction. This test was carried out independently for both participants.

Second, we tested the hypothesis that connectivity would differ between task conditions. Here, we used two metrics: whole‐brain connectivity, which is the sum of all 3003 elements in the connectivity matrix, and connectivity strength in the right sensory cortex, which is the sum of the 77 values of connectivity between the right sensory AAL region and all other regions. For both measures, we calculated eight values per condition, per participant. For each participant, we independently tested for a significant difference in connectivity using the Wilcoxon rank sum test. Again, multiple comparisons were controlled using Bonferroni correction. As a post‐hoc visualization, we averaged connectome matrices for both conditions and calculated the difference matrix (i.e., left‐handed writing minus right‐handed writing). The resulting matrix was visualized using a “glass brain”.

### Measuring robustness across runs

Neural fingerprinting assumes that every person's brain activity is unique, and consequently, a particular individual can be recognized from their brain imaging data. Based on this principle, we reasoned that—assuming our scanner provides high‐fidelity data—the functional signatures (both activity and connectivity) derived would exhibit greater similarity within participants compared to between participants. This hypothesis was tested quantitatively.

Given eight experiments, we are able to calculate 28 within‐participant comparisons per participant (i.e., 1 to 2; 1 to 3; 2 to 3, and so on) equaling 56 comparisons in total. Similarly, we are able to compute 64 possible comparisons between participants (i.e., 1 to 1; 1 to 2; 2 to 1, and so on). We could, therefore, test the hypothesis that the 56 within‐participant comparisons would, on average, be larger than the 64 between‐participant comparisons. These comparisons were quantified using the Pearson correlation coefficient for:

1. T‐statistical images. An affine transformation was applied to register all the pseudo‐T‐statistical images to the Montreal Neurological Institute standard brain. Following this, comparisons were made by vectorizing the 3D functional images and computing the Pearson correlation.
2. Connectomes. The connectome matrices derived for each experiment were vectorized, and Pearson correlation was used to quantify similarity across experiments. (Note: only data above the leading diagonal were used for correlation.)
3. Time‐frequency spectra. TFS for the left and right sensorimotor cortices were concatenated and vectorized. The Pearson correlation coefficient was used to quantify similarity across experiments.

These quantitative comparison measures were made independently for the two task conditions. For each of the three comparisons, the 64 values representing between‐participant correlations and the 56 values corresponding to the within‐participant correlations were averaged separately. The difference in mean values was then calculated as between‐participant minus within‐participant, meaning that we expected a negative value. To test statistically, we used a Monte Carlo method. An empirical null distribution was generated by randomly permuting the 120 correlation values and generating two new (sham) groups of 64 (between‐participant) and 56 (within‐participant) values. A (sham) difference metric was then computed. This process was repeated for 100,000 random permutations to generate a null distribution, and the real value (with true group labels) was compared to the null distribution.

### Triaxial reconstruction

As a final analysis, we tested the hypothesis that reconstruction of the MEG data using all three axes of measurement would be advantageous when compared to reconstruction using only radial components. To this end, we assessed the signal‐to‐noise ratio (SNR) of all signals at the channel level and statistically compared the best radially and tangentially oriented channels. SNR was measured for right‐handed writing only as the signal difference between the active and control windows and divided by the standard deviation of the signal in the active window. As before, the active window was defined as 2 s < *t* < 4 s and the control window as 5 s < *t* < 7 s, relative to stimulus onset. This gave eight measures of SNR per participant for every channel (radial and tangential). As a proxy for the total useful signal acquired across the array, SNR values were summed across channels, and the ratio of total triaxial SNR to total radial SNR was calculated. Additionally, we tested for a significant difference in SNR between the radial and tangential channels with the highest SNR, respectively, by using a nonparametric Wilcoxon rank sum test.

Finally, to assess whether triaxial reconstruction could better remove sources of external interference, we reconstructed brain activity in the 78 AAL regions using both the triaxial data (as described above) and radial‐only data. The beamformer parameters were identical in each case. We aimed to assess whether a known artifact generated at 16.6 Hz by nearby environmental effects (e.g., an air conditioning unit) could be better suppressed by the beamformer when using triaxial compared to single‐axis data. In order to visualize these reconstructed data, a TFS approach was undertaken in which data were reconstructed within 1.5‐Hz‐wide overlapping frequency bands in the 13‒30 Hz range.

## RESULTS

### Background magnetic field compensation and participant movement

Figures 2B and C show the results of background magnetic field nulling. In Figure 2B, the left‐hand chart shows the norm of the three spatially homogeneous components of the magnetic field immediately following demagnetization (left bar) and after two iterations of nulling are applied (center and right bars). The height of the bars represents the median field value across all 16 experiments, and the crosses show data from each experiment independently (participant 1 in blue and participant 2 in red). As shown, the nulling procedure reduces the median (± standard deviation) field from 3.0 ± 0.1 nT to 0.2 ± 0.1 nT. The right‐hand chart shows an equivalent measure for the five linear gradients of the magnetic field, which were reduced from 3.4 ± 0.4 nTm^−1^ to 2.0 ± 0.5 nTm^−1^. This reduction in the background magnetic field is key to enabling participant movement. The stability of the background fields between experiments and participants is also apparent in these data. Note that the initial and final fields are similar across experiments, with the exception of run 5 in participant 2 whereby the first nulling iteration failed. This is despite variations in the required head movements to sample the field between the two participants. Repeatability is also highlighted in Figure 2C, which shows the voltages applied to each of the eight coils across the eight runs in each participant. Here, the final voltages applied are similar across all experiments and demonstrate that not only the background field magnitudes but also the spatial components that define these fields were consistent over time.

**FIGURE 3:**
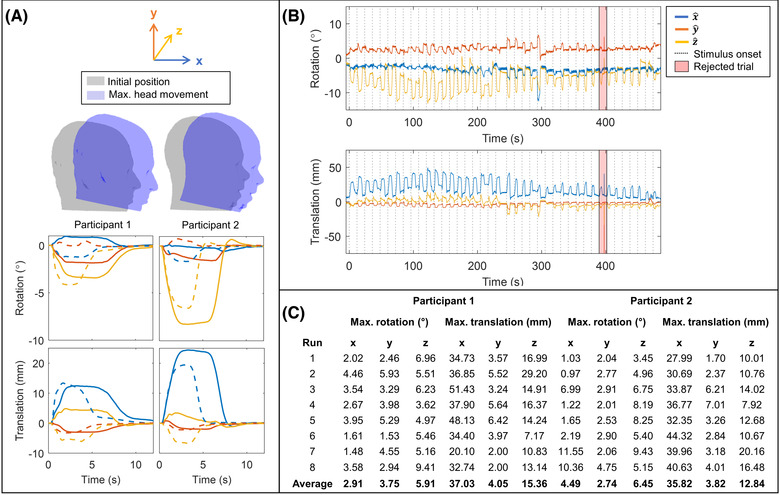
Participant movement during handwriting task. (A) Upper: Visualization of the maximum head rotation about *z* and maximum translation in *x* for each participant. Their maximum movement is shown in blue, overlaid on the initial position shown in gray. Lower: Trial‐averaged rotation (top row) and translation (bottom row), in *x*‐ (blue), *y*‐ (orange), and *z*‐directions (yellow). Average movement for left‐handed writing is given by solid lines, and right‐handed writing is shown by dashed lines. On average, participant 2 (right) moved their head more during the task than participant 1 (left). (B) Example time course showing head rotation (upper) and translation (lower) over the course of a scan. The beginning of each trial is marked by a dashed line. The red shaded region indicates a “bad trial,” where an artifact is present due to inaccuracy of the optical tracking. (C) Table of maximum rotation and translation values for both participants across all runs.

Figure 3 shows the head movement data acquired for both participants during the handwriting task. In Figure 3A, the upper plot shows a visualization of the direction and scale of movement. To perform the task, participants tended to translate forward (in *x*) and rotate to look downward (rotation about *z*). The scale of this movement is shown in the lower panel of Figure 3A, which represents trial‐averaged rotations and translations for both participants; solid lines show left‐handed writing, and dashed lines show right‐handed writing. The separate colors (blue, orange, and yellow) show translation along or rotation about the *x*‐, *y*‐, and *z*‐axes, respectively. Figure 3B shows example rotation and translation data across a single representative experiment (in participant 2), while the table in Figure 3C shows the maximum movements made in all 16 experiments. It is noteworthy that the scale of movement (up to ∼5 cm translation and 10° rotation) is such that it could not be carried out inside a conventional MEG scanner. Although conventional MEG scanners can cope with small head movements via post‐hoc correction, the scale of movement shown here would cause the participants to hit their heads on the helmet (i.e., the movement would be physically curtailed). This will be addressed further in the Discussion.

### Differences between left‐ and right‐handed writing

**FIGURE 4:**
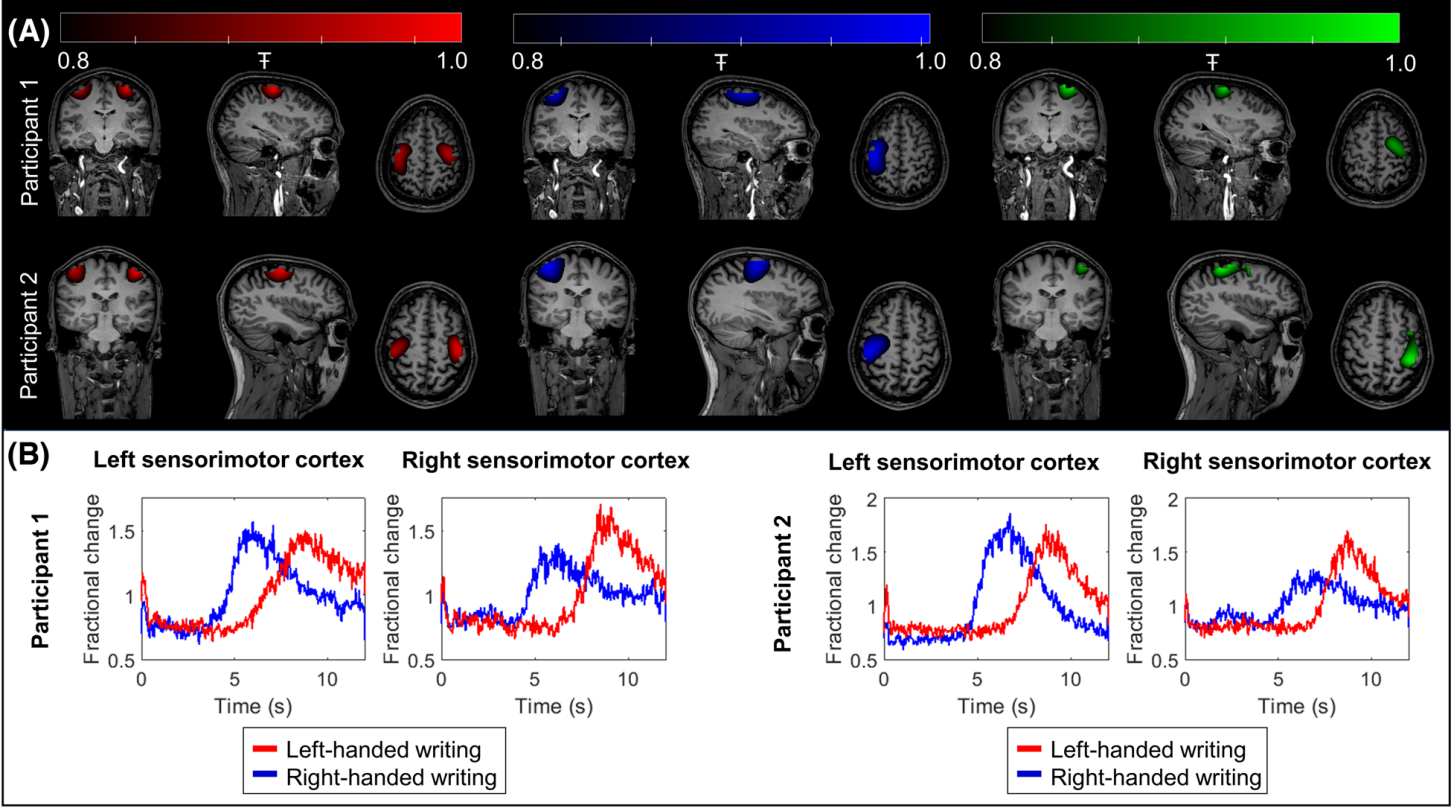
Brain function during left‐ and right‐handed writing. (A) Pseudo‐T‐statistical images, averaged across runs, thresholded at 80% of the maximum value, and overlaid on anatomical MRI. Activity during left‐handed trials is shown in red (left panel), right‐handed trials shown in blue (center panel), and the difference shown in green (right panel). Images for participant 1 are shown on the top row and participant 2 on the bottom row. For right‐ (dominant) handed writing, activity is dominant in the left motor region, whereas for left‐handed writing, a bilateral response is apparent. (B) The amplitude envelope of beta‐band activity in left and right sensorimotor cortices. Data from participant 1 are shown to the left, and participant 2 are shown on the right. For each participant, the graph to the left shows the left sensory cortex, and the plot to the right shows the right sensory cortex. In all cases, the blue traces show average (across runs) beta envelope modulation for right‐handed writing, and red shows the same for left‐handed writing.

Figure 4A shows the spatial signature of beta‐band modulation for both participants, averaged across the eight experiments. The leftmost panel (red overlay) shows left‐handed writing; the center panel (blue overlay) shows right‐handed writing, and the rightmost panel (green overlay) shows the difference between the two. In all cases, the pseudo‐T‐statistical images are thresholded at 80% of their maximum value for visualization. Note that despite participant movement throughout acquisition (Figure 3), our system was able to accurately localize beta modulation to the primary sensorimotor areas. Figure 4B shows the time courses of the beta‐band envelope extracted from the left and right sensory areas (delineated according to the AAL atlas). In each chart, beta signatures for left‐ (red) and right‐handed (blue) writing are shown. In all cases, beta power loss during movement with a rebound—above baseline—postmovement is clear. This response has been well characterized in previous studies.^39^, ^40^ Note that the rebound for left‐handed writing is comparatively delayed since participants took longer to write with their nondominant hands.

For both participants, right‐handed writing elicited a response dominated by the left motor region, whereas left‐handed writing produced a more bilateral response with beta modulation in both the left and right primary sensorimotor regions. This is shown by the functional images in Figure 4A and is also reflected in the time courses in Figure 4B, where the response in the right motor cortex is lower in amplitude for right‐handed writing (shown in blue). This lateralization was tested statistically with a summary measure of beta modulation in both left and right sensory cortices compared between task conditions. In participant 1, significantly (*p* = 0.0011; Wilcoxon rank sum test) larger beta modulation was observed in the right motor cortex for left‐handed, compared to right‐handed writing. There was no significant difference between conditions in the left motor cortex (*p* = 0.57; Wilcoxon rank sum test). In participant 2, in the right motor cortex, we again saw significantly (*p* = 0.0002; Wilcoxon rank sum test) larger beta modulation for left‐handed writing. Larger modulation in the left motor cortex was also observed in the case of right‐handed writing in this participant (*p* = 0.01; Wilcoxon rank sum test).

**FIGURE 5:**
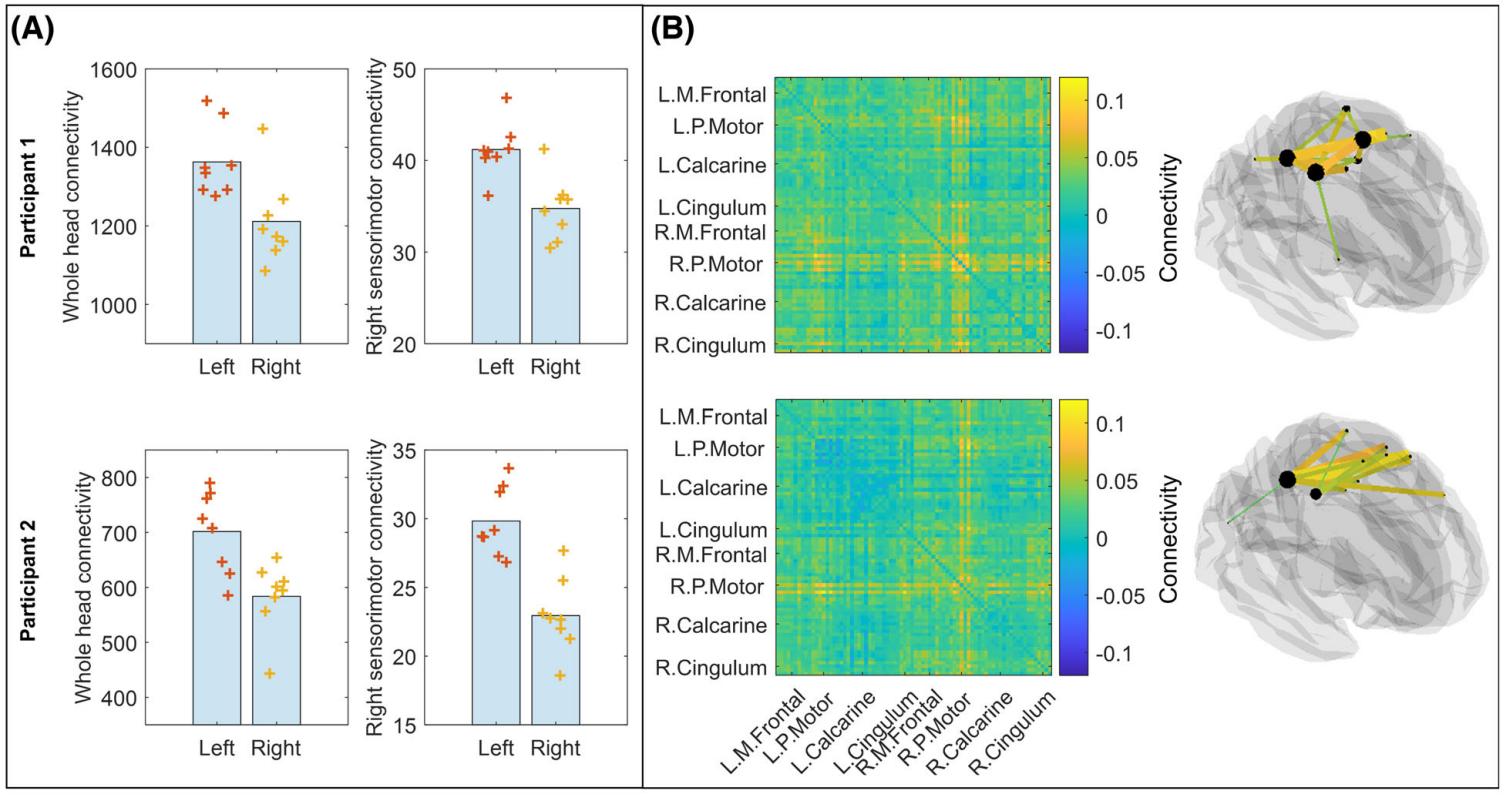
Functional connectivity. (A) Left: Whole‐brain connectivity in the beta‐band, as measured during left‐ and right‐handed trials. Results for participant 1 are shown above and participant 2 below. Right: Connectivity strength in right sensorimotor cortex measured during left‐ and right‐handed writing. For both measures, whole head and right sensorimotor cortex connectivity is significantly higher when using the left (nondominant) hand. (B) Left: Matrix representation showing the difference in connectivity across the 78 AAL regions between left‐ and right‐handed writing. Right: Glass brain visualization of a connectome for participant 1 (above) and participant 2 (below) showing the top 15% of connections. Differences are centered on the right motor cortex, and mostly interhemispheric connections are involved. Abbreviations: L. Calcarine, left calcarine sulcus; L. Cingulum, left cingulum; L.M. Frontal, left medial frontal cortex; L.P. Motor, left parietal motor cortex; R. Calcarine, right calcarine sulcus; R. Cingulum, right cingulum; R.M. Frontal, right medial frontal cortex; R.P. Motor, right parietal cortex.

Figure 5 shows the results of the functional connectivity analysis. In both participants, whole‐head connectivity (i.e., the sum of all elements in the connectivity matrix) was significantly higher for left‐handed writing compared to right‐handed writing (participant 1; *p* = 0.0047; participant 2; *p* = 0.010; Wilcoxon rank sum test). This is shown in the left‐hand panels of Figure 5A, where the bar height represents the mean connectivity across the whole head, and the crosses represent whole‐head connectivity from individual experimental runs. The results for participants 1 and 2 are shown in the upper and lower plots, respectively. This effect was amplified when looking at connectivity strength between the right sensorimotor cortex and the rest of the brain (right‐hand panels of Figure 5A) where, again, left‐handed writing resulted in significantly higher connectivity compared to right‐handed writing (participant 1; *p* = 0.0047; participant 2; *p* = 0.0006; Wilcoxon rank sum test).

Figure 5B shows the spatial signature of the connectivity differences. The matrices depict connectivity differences (left‐handed writing minus right‐handed writing) for both participants. The top 15% of connections that differ between conditions are overlaid on a glass brain for visualization. It is evident that differences are centered on bilateral connections in the motor network with significantly increased connectivity in the case of left‐handed writing.

### Measuring robustness across runs

**FIGURE 6:**
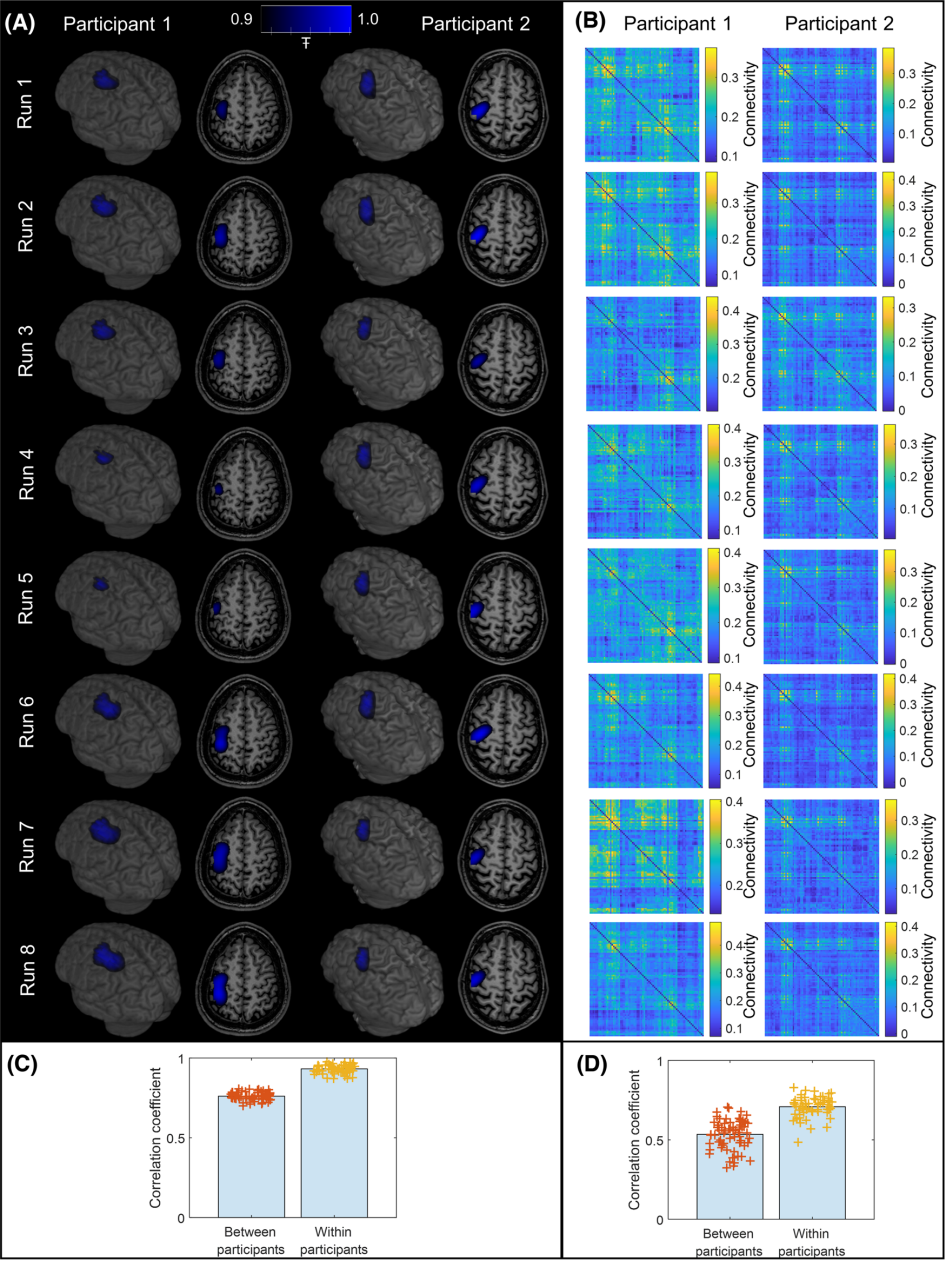
Neural fingerprinting. (A) Pseudo‐T‐statistical images for right‐handed writing in both participants were derived independently for all eight runs of the paradigm and overlaid on their brain anatomy. (B) Connectome matrices for right‐handed writing, again, were derived independently for all eight runs of the paradigm. (C) Fingerprinting analysis for the pseudo‐T‐statistical images given in (A), showing within‐ and between‐participant correlations. The bars indicate the mean correlation value, and the crosses show the individual data points. (D) Equivalent to (C) for the connectome matrices shown in (B). Differences are significant in both (C) and (D) according to our Monte Carlo‐based statistical test.

Figure 6A shows pseudo‐T‐statistical images for each of the eight experimental runs for both participants. Note the high degree of consistency across runs. Quantitatively, for the left sensorimotor cortex (during right‐handed writing), the mean Euclidean distance between the average location of peak beta amplitude modulation across all runs and each individual peak location within a run was 4 ± 2 mm for participant 1 and 5 ± 2 mm for participant 2. Equivalent values derived in the right sensorimotor cortex for left‐handed writing were 4 ± 2 mm for participant 1 and 3 ± 1 mm for participant 2.

Figure 6B shows the connectome matrices that were also derived independently from each run. In both cases, we show the case only for left‐handed writing. As with the images in Figure 6A, the connectomes are similar across runs—though there are notable differences between the two participants. These findings are formalized in the bar charts in panels C and D, which show correlation values both within‐ and between‐participants for right‐handed writing. In all cases, the bars show the mean values, while each individual data point is shown by the crosses. For the pseudo‐T‐statistical images, the correlation within participants was 0.93 ± 0.03 (mean ± standard deviation), and this was reduced to 0.76 ± 0.03 for the between‐participant correlation. For the connectomes, the correlation within participants was 0.71 ± 0.06 falling to 0.53 ± 0.09 between participants. In both cases, the difference was significant according to our Monte Carlo metric. For the time‐frequency spectra, an equivalent analysis failed to show a significant difference for within‐ and between‐participant correlations.

### Triaxial reconstruction

**FIGURE 7:**
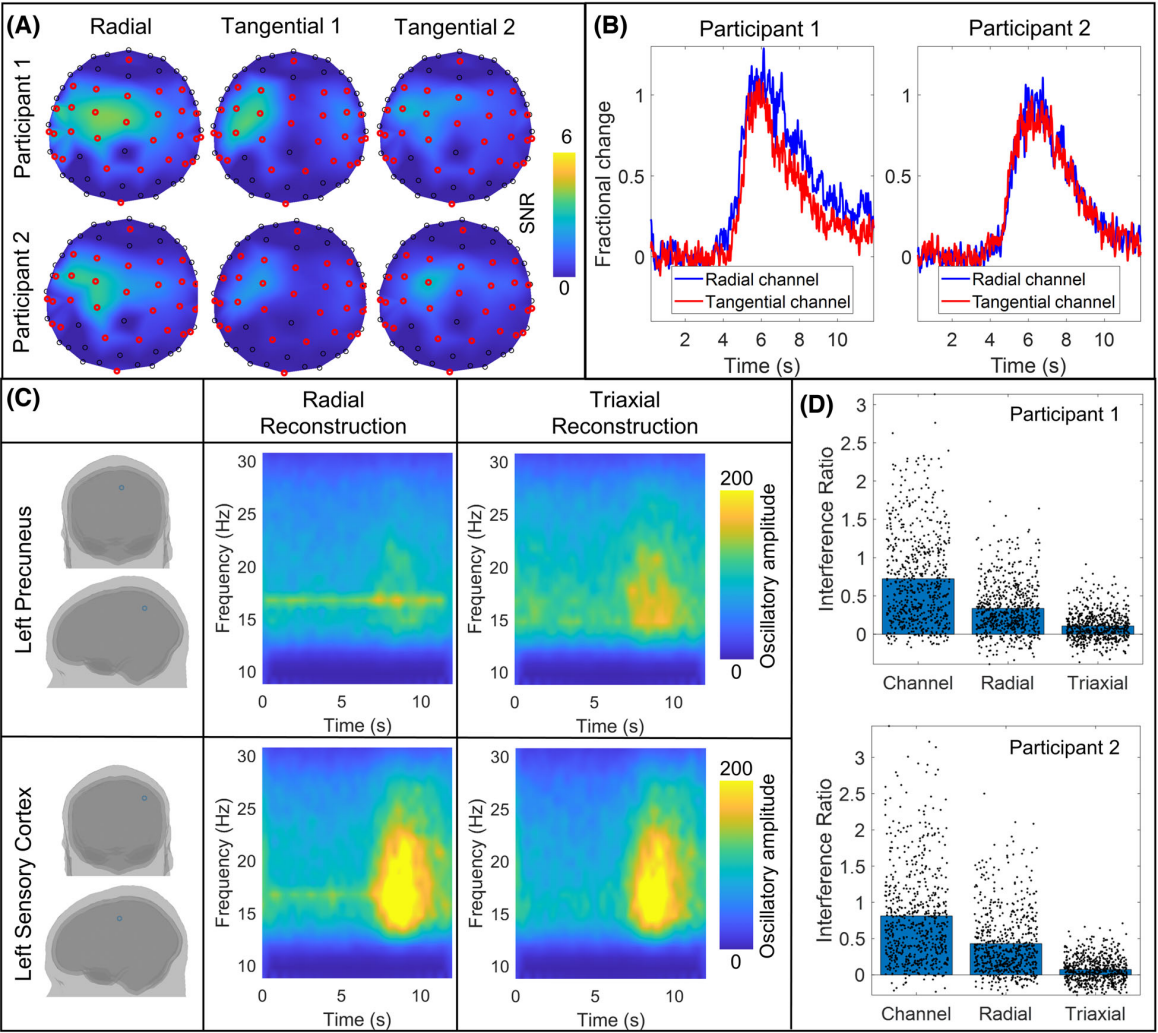
Triaxial reconstruction. (A) Topographical maps of SNR for the radial and two tangential measurements at the channel level for right‐handed writing in both participants. (B) Time courses of the beta‐band envelope were measured at the radial (blue) and tangential (red) channels with the largest SNR. (C) TFS reconstructed for beta‐band data from participant 2. Reconstructions were generated for two brain regions—the left precuneus (upper panel) and left primary sensory cortex (lower panel). The left column shows the spatial locations for the reconstruction; the center column shows radial reconstruction, while the right column shows the triaxial reconstruction. (D) Interference ratio (the ratio of the size of the interference spike, to the background [brain] activity) for both participants. The left bar shows the case for channel‐level data (the crosses indicate individual channels and runs). The center and right bars show the two different beamformer reconstructions (crosses indicate individual brain regions and runs).

Finally, Figure 7 shows the difference between single‐axis (radial) and triaxial data. Figure 7A shows a topographical representation of SNR at the channel level. Three separate maps show the cases for the radial and two tangential measurements; the orientations of the tangential measurements are defined by the OPM and helmet geometries. For participant 1, the best radially oriented channel had a mean SNR (across runs) of 6.7 ± 2.5, and the best tangentially oriented channel had an SNR of 5.8 ± 1.7. For participant 2, the best radially oriented channel had a mean SNR of 7.7 ± 2.4, and the best tangentially oriented channel had an SNR of 6.8 ± 1.0; however, there was no significant difference shown for either participant (Wilcoxon rank sum test). The mean signal from these channels is shown in Figure 7B; tangential channels are shown in red and radial channels in blue. When summing total SNR across the array, triaxial recording offered a 2.2 ± 0.2 improvement when compared to radial‐only recording for participant 1. The ratio was 2.07 ± 0.04 for participant 2.

The laboratory in which the recordings were made exhibits a known, intermittent 16.6 Hz interference source, which is likely an air‐conditioning fan. This artifact was clearly visible in 8 of the 16 recordings (four runs in each participant) based on power spectral density analysis of the raw data. For the purposes of this analysis, only those eight recordings were analyzed. Figure 7C shows an example of TFS reconstructed for beta‐band data in the left precuneus and the left primary sensory cortex. Representative data are shown from participant 2. The TFS on the right shows a triaxial beamformer reconstruction, while the TFS on the left depicts radial‐only reconstruction. Note the clear presence of the 16.6 Hz artifact when single‐axis data are used for reconstruction. This result is further characterized in Figure 7D, which shows the interference ratio. The interference ratio is defined as the difference in power spectral density between 16.6 Hz (the artifact) and its neighboring frequencies (15.5 and 17.7 Hz), divided by the power spectral density at the neighboring frequencies. Results are shown at the channel level and for both beamformer reconstructions. The bars show the mean value, while the black crosses show either individual OPM channels for the channel level or individual brain regions for the beamformer reconstructions. Data are shown independently for each participant and for all runs where the artifact was apparent. Note that the magnitude of the artifact is largest at the channel level. It is reduced by beamforming even when only using single‐axis data, but it is most effectively attenuated by triaxial reconstruction. This is in agreement with theoretical insights reported previously.^32^

## DISCUSSION

### Technical considerations

The viability of our OPM‐MEG system is demonstrated by the level of robustness of the results. The reproducibility achieved across runs was encouraging given the eight distinct experiments for each participant (data acquired across 3 days) and the extent of the head movements made by both participants throughout. In all runs, high‐fidelity metrics of beta modulation were observed, with localization identifying sensorimotor cortices with a high degree of accuracy. Quantitatively, the discrepancy in the peak locations in sensorimotor cortices between experimental runs was of order 4 mm—indicating a high degree of spatial robustness. Furthermore, differences in sensorimotor network activity and connectivity were demonstrated between left‐handed and right‐handed writing. Those differences were in strong agreement with existing literature (see below). This is again indicative of the fidelity of the acquired data. Within‐participant correlation of the pseudo‐T‐statistical images (another indicator of spatial accuracy) was 93%, and the within‐participant correlation of the connectomes, which is a marker of both spatial and temporal signal acuity, was 71%. It is also noteworthy that for both pseudo‐T‐statistical images and connectome matrices, within‐participant correlations were significantly greater than between‐participant measures. These correlations demonstrated that the differences between individuals were larger than the differences across runs in the same participant—in accordance with results reported previously.^54^ We interpret this as an additional marker of robustness. Overall, the evidence points to our 90‐channel system offering an excellent means to interrogate brain electrophysiology during a naturalistic task. However, while repeatability was high across runs, it was not perfect. It is unknown whether the remaining discrepancies are due to instrumental instability, genuine differences in brain activity between runs (due to, e.g., the time of day and fatigue), or a mixture of the two.

Precise control of the background magnetic field is a critical factor in successful OPM‐MEG operation. This is because in the SERF regime, which our sensors exploit, OPM output is only linear with the applied field around zero‐field. If background magnetic fields begin to increase, the effective sensor gain is diminished (i.e., for the same change in the field, the sensor output will be lower). Prior to field nulling, but after demagnetization, the background field in the MSE was ∼3 nT (Figure 2B). This is much lower than the ambient field found in most MSEs used for MEG where we might expect fields between 10 and 70 nT. However, even at 3 nT, we estimate that if a 90° rotation of the head (hence a 90° rotation of the OPMs) was carried out, some sensors would experience a gain change of ∼3.8%. Such changes are known to affect the quality of MEG reconstruction.^13^ However, following nulling, the background field was reduced to 0.2 nT meaning an equivalent 90° rotation would generate only a 0.018% gain change. For the scale of a movement carried out during our handwriting paradigm (∼5 cm translation and ∼10° rotation—see Figure 3), sensor gain changes would have been of order 0.2% had experiments been carried out with no nulling. However, with nulling, these gain changes were reduced to a maximum of ∼0.0005%. In sum, the nulling procedure, coupled with dynamic stabilization, which keeps the background field stable over the duration of the experiment, ensures that the OPM output remains linear—providing a faithful representation of the magnetic field even in the face of large participant movements. This is a critical feature of our system. An important consideration is that while nulling resulted in a 0.2 nT remnant magnetic field on average, in many instances, better performance was achieved, with the best being 0.1 nT. The differences in performance between runs are not yet understood but may relate to the accuracy of tracking, small movements of the reference array, or the calibration accuracy of the coil system. In principle, if such errors can be corrected, there is no fundamental reason why background fields cannot be driven even lower; while this would have relatively little impact on gain changes, which are already negligible, it will further reduce low‐frequency artifact caused by movement and hence elevate SNR.

Our array was constructed using triaxial sensors. This contrasts with most conventional MEG systems and many OPM‐MEG systems,^34^, ^45^ which measure only the radial component of neuromagnetic field or field gradient. There are numerous advantages to the triaxial measurement that should be explored. First, triaxial sensors generate three times more measurements compared to a single‐axis system. This does not equate to three times more signal since, in the tangential axes, the magnitude of the field from the brain is smaller in comparison to the radial axes.^12^, ^31^, ^32^ Nevertheless, experimental measurements (Figure 7) showed that triaxial recording offered twice the total useful signal (summed SNR across the array) compared to single‐ (radial) axis recordings. On average, the highest SNR was 6.3 for tangentially orientated sensors compared to 7.2 for radially oriented sensors. The fact that the tangential measure was not significantly smaller than the radial measure is likely the result of variability across experiments in signals at the channel level due largely to the different positions of the helmet for each run. Nevertheless, the comparable SNR shows that a useful signal is detected in the tangential axes, and this additional signal will offer significant benefits for source localization.^32^ Importantly, though not shown explicitly here, when a limited sampling array is used, the uniformity of coverage is also improved by triaxial measurement^31^, ^33^—a finding that is of critical importance when imaging infants using scalp‐mounted sensors.

Second, triaxial sampling enables better separation of magnetic fields originating from inside the head (the MEG signal) from fields originating outside (interference). This, in turn, enables improved rejection of external interference. This finding is not new. The previous work^55^ demonstrated that the addition of tangentially oriented SQUID sensors to a conventional system enabled the better separation of interference sources from the MEG signal. Likewise, our own work has theoretically shown how beamforming can be used to exploit the additional information on interfering magnetic fields provided by a triaxial array.^32^ Here, this effect was investigated experimentally via single‐axis and triaxial beamformer reconstruction of the same data. Such analysis is, to an extent, confounded because of the total number of sensors and orientation sensitivity changes. Nevertheless, the data given in Figure 7 demonstrate how the effect of a known source of external interference on MEG data can be made negligible by the use of triaxial reconstruction. This is an extremely important consideration for OPM‐MEG system design. Conventional MEG systems are often formed from gradiometers, which measure the rate of change of magnetic field over space rather than the absolute field value. In the simplest form, gradiometers measure the field change between a radially oriented coil close to the head and another radially oriented coil around 5 cm away. The second coil will measure similar interference, which tends to be spatially uniform but will be less sensitive to the field from the brain, which decays rapidly with radial distance. The result is an overall metric that retains the brain signal but rejects interference. It is possible to construct a similar system of gradiometers based on OPMs^34^ and thus control interference in the same way. However, the need for distal sensors would make an OPM helmet bulky, and wearable devices would likely become impractical. A different scheme may use planar gradiometers where the field gradient is measured tangentially rather than radially. Indeed, this has been demonstrated successfully;^7^ however, the physical size of the gradiometers then becomes an issue, and either array density is compromised (i.e., fewer planar gradiometers can be arranged on the head) or the baseline (the distance over which gradient is measured) becomes small, limiting depth sensitivity. For these reasons, control of background interference with OPMs is challenging, and the use of conventional gradiometric solutions poses problems, particularly if we wish to maintain wearability. Triaxial sensitivity and the advantages that it brings thus represents a significant and viable method of interference rejection. Importantly, while here we show this effect via beamforming, there are many other interference rejection metrics (e.g., Ref. 56) that would likely benefit from the additional information provided by triaxial measurements.

In addition to the above considerations, there are important technical advantages for a triaxial OPM. First, since triaxial sensitivity enables measurement of the full field vector, a *complete and unambiguous calibration* of the sensor, including its gain and orientation sensitivity, can be carried out. Specifically, three separate pulses of the magnetic field can be applied using the on‐sensor coils. For example, any signal generated on the *y*‐axis measurement from an *x*‐axis pulse can then be characterized and corrected in software. This orthogonalization implemented in the present sensors means that the OPM will provide a true estimation of not only field magnitude but also direction. This calibration can be used to eliminate crosstalk both between sensors (i.e., perturbations in measurements at one sensor due to the presence of a neighboring sensor) and crosstalk between axes within the same sensor—a significant advantage compared to single‐ or dual‐axis sensing. Finally, triaxial sensitivity is essential for closed‐loop operation in practical use. Unlike solid‐state vector magnetometers, OPM sensitivity, calibration, and directionality in the *z*‐direction depend not only on the magnetic field in the *z*‐direction but equally strongly on the magnetic field in the *x*‐ and *y*‐directions (cross‐axis projection errors^57^). With triaxial sensitivity, a closed‐loop operation can stabilize the internal magnetic field of an OPM in all three directions,^58^ thereby increasing the dynamic range, stabilizing the sensor gain, and making OPMs tolerant to changes in the magnetic field in any direction.

This said, rather than use closed‐loop operation on all sensors individually, we chose to control the linearity of the response by using the biplanar coils and dynamic stabilization. This nulls the background magnetic field across the OPM array as a whole, ensures a linear response, and negates the need for closed‐loop operation.

OPM‐MEG is still in development, and there are several limitations of the current system that should be acknowledged. First, brain coverage in the current system was limited because only 30 triaxial OPMs were available. However, there is no reason why we cannot increase the number of sensors to provide whole‐brain coverage. Indeed, previous work^45^, ^54^ showed that an OPM‐MEG system with 50 radial‐only channels performed well compared to a state‐of‐the‐art cryogenic system. We strongly believe that a similar number of triaxial sensors would give both whole‐brain coverage and better performance. Second, the noise floor of OPMs is currently higher than the noise floor of SQUIDs. Therefore, future work on sensitivity may facilitate further improvements in results. Miniaturized OPMs are still a recent development, and their performance has improved significantly over recent years; there is no fundamental reason that OPMs cannot surpass the sensitivity of SQUIDs. Indeed, it is likely that improved lasers may offer a direct route to this goal. Third, a significant challenge is the heat generated by the sensors during operation. In the current system, a judicious helmet design kept the temperature on the scalp comfortable. However, as systems move toward higher numbers of sensors, greater amounts of heat will dissipate with the potential to become uncomfortable for the participant. Future OPM‐MEG systems may then require active cooling within the OPM helmet.^59^ Finally, although our magnetic field compensation approach successfully provided a low‐field environment, the location of the nulled volume was at the center of the room and around 0.5 × 0.5 × 0.5 m^3^ in volume. This means that while head movements can be carried out with minimal effect on sensor operation, larger movements (e.g., a participant walking) remain prohibited. The development of new “reconfigurable” coils to generate nulled volumes at any location inside the MSE,^60^ and a drive to a similarly low remnant field, therefore, remains a critical area of development.

### Neuroscientific findings

This study was conducted with the aim of evaluating the fidelity of our OPM‐MEG system rather than to investigate the neural substrates underlying handwriting. Nevertheless, our findings warrant some discussion. We measured fluctuations in beta activity that were localized to primary sensory and motor cortices during handwriting. As might be expected, when writing with the dominant hand, beta modulation was lateralized with the strongest in the contralateral cortex. However, a more bilateral response was observed when writing with the nondominant, left hand—a finding that was significant in both participants independently. A secondary finding showed increased bilateral sensorimotor connectivity during left‐handed, compared to right‐handed, writing. Spatial analysis showed increased interhemispheric connectivity between the left and right motor regions, which is consistent with the idea that a bilateral network of brain areas was recruited to perform the task with the nondominant hand. These observations are in strong agreement with previous literature. For example, in an early functional magnetic resonance imaging (fMRI) study, Kim et al.^61^ showed a hemispheric asymmetry in the activation of the motor cortex during simple movements. Likewise, Singh and colleagues^62^ used fMRI to show that in right‐handed participants, ipsilateral activation was significantly greater when participants carried out a task with their nondominant, compared to their dominant, hand. While these studies report the blood oxygenation level‐dependent (BOLD) hemodynamic response, which is distinct from the neurophysiological response measured in MEG, there is a known link between beta oscillations and BOLD;^63^ therefore, we would expect similar asymmetries to be apparent in beta modulation.

Handwriting itself is a complex procedure that evokes activity across a number of brain areas. Here, we studied the sensorimotor network, which is integrally involved but not specific to handwriting. A meta‐analysis performed by Planton et al.^64^ demonstrated the involvement of a much larger network, including the left superior frontal sulcus, middle frontal gyrus, left intraparietal sulcus, superior parietal area, and right cerebellum. All of these brain areas were said to be writing‐specific. Other areas include those associated with linguistic processes, such as the posterior/inferior temporal cortex. Our study was limited by coverage since only 30 sensors were available. We, therefore, chose to focus on the left and right motor regions. In addition, we focused primarily on beta‐band oscillations, which are well known to be the dominant rhythm within the sensorimotor system—although other brain rhythms are also known to be associated with motor control.^65^ For this reason, future studies of this type of paradigm should employ a sensor array that facilitates whole‐head coverage to increase sensitivity to other regions and look to investigate both oscillatory modulation and connectivity across a broader range of frequencies.

Finally, we note the potential of OPM‐MEG to measure brain function during naturalistic tasks. Importantly, handwriting has been carried out across a variety of scanning environments, including positron emission tomography,^64^ fMRI,^64^ and conventional MEG,^65^ which shows that it is possible to carry out tasks of this nature even in a restricted scanning environment. However, as demonstrated by data in Figure 3, *natural* handwriting involves movements of the head that would be extremely challenging to carry out in conventional systems. For example, in fMRI, participants are typically supine with movement severely restricted, and spin history effects can modulate the BOLD signal acquired over time. Participant movement is also often, but not always, physically restricted by the presence of a dedicated head radiofrequency coil. In conventional MEG, any motion of the participant relative to the fixed sensor array causes changes in signal amplitude, SNR, and field topography. As with fMRI, algorithms to correct such artifacts are available (e.g., Refs. 56, 65–71). But even with movement compensation, successful MEG measurement relies on the participant's head remaining inside the helmet and places a hard limit on permitted head movements. Figure 3 demonstrates that the scale of movement evoked by our handwriting task could not have been carried out in a conventional MEG scanner without the participant's head coming into contact with the helmet. Conversely, OPM‐MEG has enabled a scale of movement in which natural handwriting can be achieved. Moreover, these results pave the way for more expansive paradigms enabling a greater degree of head movement, which has the potential to facilitate a new generation of neuroscientific study.

## CONCLUSION

OPM‐MEG is an emerging functional imaging technique offering greater flexibility to design new naturalistic neuroscientific paradigms and scan cohorts of individuals who struggle to comply with conventional imaging environments. In addition, it promises high sensitivity and spatial resolution since sensors require no cryogenic cooling and can be positioned closer to the brain than the SQUID‐based sensors used in conventional MEG. However, the technology is new, and the optimum design for a viable OPM‐MEG system is far from settled. Here, we have constructed and demonstrated a novel 90‐channel OPM‐MEG system based on triaxial OPM sensors operated in an optimized low‐field environment. Results show that our background magnetic field compensation reduced the field inside the MSE to a level of around 0.2 nT, enabling participant movement with minimal effect on sensor operation. Further, in agreement with theoretical studies, our triaxial array offered not only high‐fidelity reconstruction of electrophysiological function (i.e., peak activation in sensorimotor cortices localized within ∼4 mm across repeat scans) but also significant advantages for characterization and rejection of external interference compared to what would be achieved with single‐axis (radial) OPMs. We were able to record data during a naturalistic handwriting paradigm with results showing significant differences in the spatiotemporal profile of activity and connectivity for left‐handed and right‐handed writing. These results are in strong agreement with previous studies. Overall, our study highlights the unique potential of OPM‐MEG as a high‐performance method for functional neuroimaging.

## AUTHOR CONTRIBUTIONS

M.R. contributed to the study conceptualization, data collection, data analysis, data interpretation, and writing and review of the paper. M.J.B. contributed to the study conceptualization, data analysis, data interpretation, and writing and review of the paper. E.B. and N.H. contributed to the study conceptualization, data collection, data interpretation, and paper review. R.H. contributed to data analysis, data interpretation, and paper review. J.O., N.R., J.L., and L.R. contributed to data collection and paper review. R.B. contributed to the study conceptualization and paper review. V.S. contributed to study conceptualization, data collection, and paper review.

## COMPETING INTERESTS

E.B. and M.J.B. are directors of Cerca Magnetics Limited, a spin‐out company whose aim is to commercialize aspects of OPM‐MEG technology. E.B., M.J.B., R.B., N.H., and R.H. hold founding equity in Cerca Magnetics Limited, and R.B., N.H., and R.H. sit on the scientific advisory board. E.B. is on the scientific advisory board of MYndSpan Ltd. The authors are involved in U.K. patent application numbers GB2015427.4, GB2106961.2, and GB2108360.5; all of which relate to OPM‐MEG. V.S. is the founding director of QuSpin Inc., a commercial entity selling OPMs. J.O. is an employee of QuSpin. V.S. and J.O. are involved in U.S. patent number US10775450B1, which relates to triaxial OPMs.

### PEER REVIEW

The peer review history for this article is available at: https://publons.com/publon/10.1111/nyas.14890.
